# Utilization of a Conidia-Deficient Mutant to Study Sexual Development in *Fusarium graminearum*

**DOI:** 10.1371/journal.pone.0155671

**Published:** 2016-05-13

**Authors:** Hokyoung Son, Jae Yun Lim, Yoonji Lee, Yin-Won Lee

**Affiliations:** 1 Center for Food and Bioconvergence, Seoul National University, Seoul, Republic of Korea; 2 Department of Agricultural Biotechnology, Seoul National University, Seoul, Republic of Korea; Soonchunhyang University, REPUBLIC OF KOREA

## Abstract

Transcriptome analysis is a widely used approach to study the molecular mechanisms underlying development and the responses of fungi to environmental cues. However, it is difficult to obtain cells with a homogeneous status from the sexually-induced culture of the plant pathogenic fungus *Fusarium graminearum*. In this study, we provided phenotypic and genetic evidence to show that the current conditions applied for perithecia induction inevitably highly induced asexual sporulation in this fungus. We also found that hundreds of genes under the control of the conidiation-specific gene *ABAA* were unnecessarily upregulated after perithecia induction. Deletion of *ABAA* specifically blocked conidia production in both the wild-type strain and sexually-defective mutants during sexual development. Taken together, our results suggest that the *abaA* strain could be used as a background strain for studies of the initial stages of perithecia production in *F*. *graminearum*. Further comparative transcriptome analysis between the *abaA* mutant and the sexually-defective transcription factor mutant carrying the *ABAA* deletion would contribute to the construction of the genetic networks involved in perithecia development in *F*. *graminearum*.

## Introduction

Fusarium head blight (FHB) is an important and widespread disease of major small grains. *Fusarium graminearum* is the primary causative agent of FHB worldwide [1]. In addition to yield and quality losses, the importance of FHB lies in the accumulation of harmful mycotoxins [2]. FHB epidemics continue to occur throughout the world in accordance with recent emerging fungal diseases in animals and plants [3,4]. Because few resistant cultivars and fungicides with effective applications and competitive prices are available, FHB remains difficult to control [4]. Therefore, understanding the molecular mechanisms involved in the *F*. *graminearum* life cycle is required to develop effective strategies to control FHB.

*F*. *graminearum* uses conidia (asexual spores), ascospores (sexual spores), and hyphal fragments as disease inocula [5]. Whereas conidia and hyphal fragments are dispersed short distances by wind or rain-splash, ascospores in the perithecia are forcibly discharged into the air and can move several kilometers; therefore, ascospores function as the primary inocula instead of conidia [6,7]. Moreover, perithecia and perithecia-associated mycelia are known to play roles in the overwintering process [8]. Sexual reproduction ensures genetic diversity in the population, which provides a capacity for adaptability towards host plants [9,10].

Sexual reproduction of *F*. *graminearum* is accompanied by distinct alterations in cellular differentiation processes and metabolism [11,12]. Therefore, fine-tuned temporal and spatial gene regulation is necessary for proper fertilization, fruiting body formation, ascospore maturation and discharge. Because numerous metabolic and major signal transduction pathways are closely linked to these processes, disruption of the genes involved in these processes commonly results in defects in various steps of *F*. *graminearum* sexual development [13,14]. For instance, a genome-wide functional analysis of transcription factors and kinases identified hundreds of genes involved in sexual development and contributed to the comprehensive understanding of the genetic networks involved in *F*. *graminearum* sexual reproduction [15,16].

Transcriptome analysis utilizing the next-generation sequencing-based RNA-seq is a powerful approach that can be used to study the molecular mechanisms involved in various developmental stages and the responses of fungi to specific environmental stresses. Additionally, comparative transcriptome analysis between the wild-type strain and null mutants enables the characterization of novel genes or genetic pathways that are under the control of upstream signal transduction pathways or specific transcription factors. Recent advances in next-generation sequencing technologies enabled researchers to perform transcriptome analyses even in non-model filamentous fungi [17].

One of the critical factors that determines the success of transcriptome analysis is the extraction of mRNA from cells with a homogeneous status to exclude false-positive results. In filamentous fungi, there has been a limit to the ability to obtain fungal cells with the same physiological and/or developmental status because few conditions are available for the simultaneous induction of specific developmental stages. Recent advances in RNA-seq technology enabled transcriptome analysis with a minute amount of sample, such as microdissected tissues and even a single cell [18,19]. However, most high-tech methods are difficult to apply to filamentous fungi due to the limited accessibility of equipment and technological limitations.

It is also very important to obtain morphologically and/or physiologically distinct cells for the study of sexual development in filamentous fungi such as *F*. *graminearum*. However, mycelial growth and asexual sporulation commonly occur, and only a small portion of hyphal cells differentiate into fruiting bodies after sexual induction. Therefore, the development of specific conditions to suppress other developmental processes or dissecting methods to obtain specific cells/tissues are required to obtain a high quality transcriptome during sexual development in filamentous fungi.

AbaA is a well-known central regulator for conidiation and the distinct genetic pathway, BrlA-AbaA-WetA, orchestrates each step of conidiogenesis in *Aspergillus nidulans* and *A*. *fumigatus* [20,21]. In a previous study, we identified and functionally characterized the *ABAA* ortholog, which was previously thought to be absent in *F*. *graminearum* [22]. We found that AbaA is specifically required for phialide formation and function, and that AbaA exclusively localized to nuclei during conidiogenesis. Subsequent studies demonstrated that the AbaA-WetA pathway of *A*. *nidulans* is conserved in *F*. *graminearum* [23]. WetA is under control of AbaA and is required for phialide function and conidia maturation.

In this study, we found that the current condition for *in vitro* sexual induction highly induced asexual sporulation and resulted in the unnecessary expression of conidiation-related genes that disturbed proper transcriptome analysis in *F*. *graminearum*. Based on our previous genetic results and bioinformatics verification, we propose that the conidia nonproducing mutant, *abaA*, is useful for studies of *F*. *graminearum* sexual development.

## Materials and Methods

### Fungal strains and media

The wild-type strain Z-3639 and transgenic strains derived from this strain were used in this study (Table 1). The conidia nonproducing mutant Δ*abaA* and transcription factor mutants were described in previous studies [15,22]. All strains were stored as conidia and mycelia in a 30% glycerol solution at -80°C. Conidia production was induced on yeast malt agar (YMA) as previously described [24]. All other media used in this study were prepared as described in the *Fusarium* laboratory manual [25].

**Table 1:** *F. graminearum* strains used in this study.

Strain	Genotype	Source or reference
Z-3639	*Fusarium graminearum* wild-type	[26]
Δ*abaA*	Δ*abaA*::*GEN*	[22]
Δ*mat2*	*∆mat1-2*::*GFP-HYG*	[27]
HK167	Δ*mat1-2*::*HYG* Δ*abaA*::*GEN*	Δ*mat2* × Δ*abaA*
Δ*gzp53l005*	Δ*gzp53l005*::*GEN*	[15]
Δ*gzzc183*	Δ*gzzc183*::*GEN*	[15]
Δ*gzzc258*	Δ*gzzc258*::*GEN*	[15]
Δ*Fgnot3*	Δ*Fgnot3*::*GEN*	[15,28]
Δ*gzzc302*	Δ*gzzc302*::*GEN*	[15]
HK327	Δ*gzp53l005*::*GEN* Δ*abaA*::*GEN*	HK167 × Δ*gzp53l005*
HK328	Δ*gzzc183*::*GEN* Δ*abaA*::*GEN*	HK167 × Δ*gzzc183*
HK329	Δ*gzzc258*::*GEN* Δ*abaA*::*GEN*	HK167 × Δ*gzzc258*
HK330	Δ*Fgnot3*::*GEN* Δ*abaA*::*GEN*	HK167 × Δ*Fgnot3*
HK331	Δ*gzzc302*::*GEN* Δ*abaA*::*GEN*	HK167 × Δ*gzzc302*

### Sexual crosses

Sexual reproduction was induced by removing 5-day-old aerial mycelia with the back of the surgical blade (surgical blade #11; Feather Safety Razor, Osaka, Japan) and 2.5% sterilized Tween 60 solution for the self-cross [1]. For the outcrosses, the heterothallic ∆*mat2* strain was fertilized with 1 ml of a conidial suspension (10^5^ conidia/ml) from fertilizing parents [27]. All sexually-induced cultures were incubated under near UV light (365 nm wavelength; HKiv Import & Export Co., Ltd., Xiamen, China) at 25°C.

### RNA-seq and bioinformatics analysis

The RNA-seq analysis was performed as previously described [22]. Total RNA was extracted using an Easy Spin Total RNA Extraction Kit (iNtRON Biotech, Seongnam, Korea). RNA-seq libraries were constructed using the Illumina TruSeq^TM^ RNA sample prep kit (Illumina, San Diego, CA USA) with no modifications to the standard low-throughput protocol. Samples were run on an Illumina HiSeq2000 instrument using the reagents provided in the Illumina TruSeq paired-end (PE) cluster kit V3-cBot-HS and the TruSeq SBS kit v3-HS (200 cycles).

The data discussed in this publication have been deposited in NCBI's Gene Expression Omnibus [29] and are accessible through GEO Series accession number GSE46133 (http://www.ncbi.nlm.nih.gov/geo/query/acc.cgi?acc=GSE46133) and GSE79532 (http://www.ncbi.nlm.nih.gov/geo/query/acc.cgi?acc=GSE79532). Raw RNA-seq data from 2 h and 24 h after sexual induction (WT-2 h and WT-24 h) were obtained in a previous study [30] and realigned into the genome obtained from the *Fusarium graminearum* Genome Database [31] for the comparative transcriptome analysis.

Alignments were generated with BWA [32] using the *F*. *graminearum* genome [31] and the htseq-count script in the HTSeq package was used to compute the counts per gene [33]. Genome-wide transcript levels were quantified in reads per kilobase of exon per million mapped sequence reads (RPKM) [34]. Genes with maximum RPKM × 100 values below 10 were removed from the analysis. Three RNA-seq replicates were performed for each sample and mean values of them were used to calculate fold changes. Differentially expressed genes were identified based on fold change values. The time-course expression patterns during conidiation were clustered into 10 groups using the R package *Mfuzz* with default setting, which performs fuzzy c-means clustering [35].

## Results and Discussion

### Conidia production after sexual induction in *F*. *graminearum*

**Fig 1:**
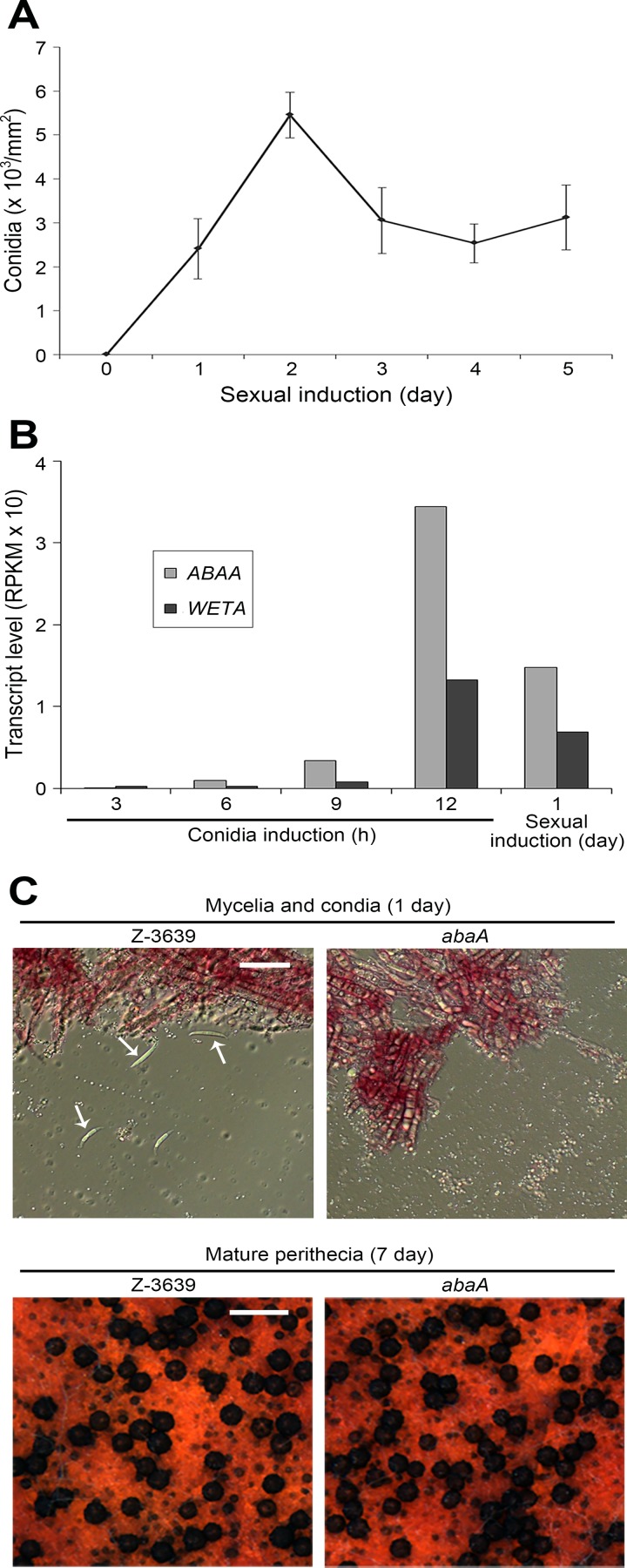
Induction of asexual sporulation during perithecia production in *F*. *graminearum*. (A) Conidia production after sexual induction. The number of conidia (conidia/mm^2^) was counted 0–5 days after sexual induction in the wild-type strain. (B) *ABAA* and *WETA* transcript levels during asexual and sexual reproduction. The transcript levels were quantified in reads per kilobase of exon per million mapped sequence reads (RPKM). (C) Mycelia and perithecia produced from the wild-type and *abaA* mutant strains. Scale bars represent 50 μm and 500 μm for the upper and below pictures, respectively. Arrows indicate mature conidia.

Sexual and asexual reproduction are commonly induced together in various *Fusarium* species, including *F*. *graminearum* [1]. To exclude the effects of conidiation from the transcriptome analysis during perithecia production in *F*. *graminearum* and *F*. *verticillioides*, in a previous study the surface of the agar medium was washed and visible perithecia were picked for RNA extraction [30]. Similarly, we observed that vigorous conidia production occurred in the current condition applied for *F*. *graminearum* sexual reproduction (Fig 1A and 1C). The conidia number peaked 2 days after sexual induction and was highly maintained during the sexual developmental stages. The results demonstrated that transcripts accumulated during conidiation; therefore, the transcripts stored within conidia should be included in the transcriptome of sexually-induced fungal cultures, especially during the initial stages (~ 2 days). Thousands of genes exhibit altered expression during *F*. *graminearum* conidiation [22,23], and dormant conidia have been known to store many transcripts in filamentous fungi [36,37].

To analyze the transcriptome during conidiogenesis and the initial stage of perithecia production, we performed RNA-seq 3 and 9 h after conidia induction and 1 day after sexual induction in the *F*. *graminearum* wild-type strain Z-3639. The transcriptomes 6 and 12 h after conidia induction were obtained in a previous work [22]. Because all of the RNA-seq experiments were performed using the same platform (GEO platform ID, GPL17573) and normalized together, the RPKM values of each sample could be compared.

We investigated the expression of the conidiation-specific genes *ABAA* and *WETA* during conidiogenesis (Fig 1B). Our previous results showed that the AbaA-WetA pathway was specifically involved in phialide formation and function in *F*. *graminearum* [22,23]. In accordance with our previous results, *ABAA* transcripts began to increase 6 h after conidia induction and peaked at 12 h. *WETA* was expressed later than *ABAA*, confirming the involvement of the AbaA-WetA pathway for conidiation in this fungus. Transcript amounts of both *ABAA* and *WETA* 1 day after sexual induction were approximately half of those detected 12 h after conidia induction (Fig 1B). Taken together, we concluded that the present condition for sexual reproduction also highly induced asexual sporulation in *F*. *grainearum*.

### Induction of conidiation-related genes during the initial stage of perithecia development

We investigated the extent of the distortion of the perithecia production transcriptome by unintended conidia induction in *F*. *graminearum*. *ABAA* deletion resulted in a complete lack of conidia production but normal sexual development (Fig 1C). Moreover, *ABAA* deletion did not affect any other fungal developmental stages in *F*. *graminearum*, including vegetative growth and virulence [22]. To analyze the *abaA* mutant transcriptome during the initial stage of perithecia development, we performed RNA-seq 1 day after sexual induction of the *abaA* mutant and compared the results with other transcriptome data (S1 Table). Overall, 933 and 472 genes were upregulated and downregulated, respectively, in the wild-type strain compared to the *abaA* mutant, showing that a profound alteration in gene expression occurred when conidiation was blocked during sexual development.

**Fig 2:**
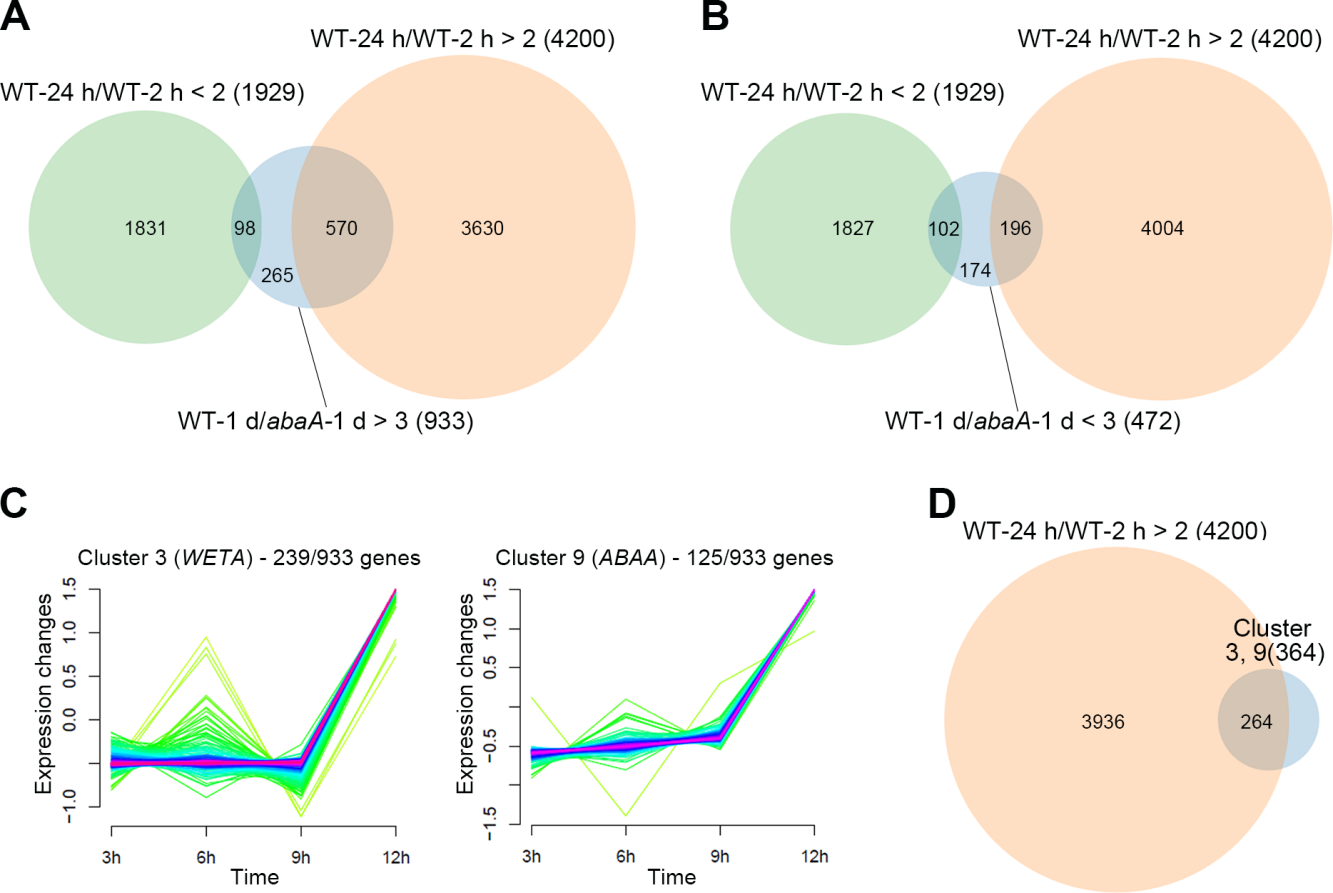
Induction of conidiation-related genes during the initial stage of sexual development. (A) Venn diagrams illustrating the overlap between upregulated (A) or downregulated (B) wild-type-specific genes (blue circle, WT-1 d/*abaA*-1 d) and differentially expressed genes after sexual induction in the wild-type strain (green circle for downregulated genes, WT-24 h/WT-2 h < 2; orange circle for upregulated genes, WT-24 h/WT-2 h > 2). (C) Expression profiles of clustered groups including the conidiation-specific genes *WETA* and *ABAA*. Fuzzy clustering categorized 933 upregulated wild-type-specific genes into 10 groups. The genes included in groups 3 and 9 showed similar expression patterns during conidiation with *WETA* and *ABAA*, respectively. (D) The Venn diagram illustrating the overlap between the *WETA*- and *ABAA*-related genes (blue circle) and the upregulated genes after sexual induction in the wild-type strain (orange circle). Wild-type-specific genes (WT-1 d/*abaA*-1 d) represent differentially expressed genes in the wild-type strain compared to the *abaA* mutant 1 day after sexual induction. Genes that were differentially expressed 24 h after sexual induction compared to the uninduced condition (2 h) represent differentially expressed genes after sexual induction [30].

We compared genes that were differentially expressed due to the *ABAA* deletion (WT-1 d/*abaA*-1 d > 3 or < 3) with genes that exhibited altered expression during the initial stage of perithecia development in the wild-type strain (WT-24 h/WT-2 h > 2 or < 2). Raw RNA-seq data 2 h and 24 h after sexual induction were obtained from a previous study [30] and realigned for our analysis (S1 Table). Overall, 4200 and 1929 genes were upregulated and downregulated, respectively, 24 h after sexual induction compared to the uninduced condition (2 h). More than half of the upregulated genes in the wild-type strain compared to the *abaA* mutant exhibited simultaneous increases in expression during the initial stage of sexual induction (WT-1 d/*abaA*-1 d > 3 and WT-24 h/WT-2 h > 2; 570/933); however, a small portion of genes overlapped with genes that were downregulated during perithecia development (WT-1 d/*abaA*-1 d > 3 and WT-24 h/WT-2 h < 2; 98/933) (Fig 2A). The result suggests that approximately 14% (570/4200) of the sexually-induced genes may be related to conidiation but not perithecia development in *F*. *graminearum*. Among the downregulated genes in the wild-type strain compared to the *abaA* mutant, a relatively high proportion of genes were repressed during the initial stage of sexual induction (WT-1 d/*abaA*-1 d < 3 and WT-24 h/WT-2 h < 2; 102/472) (Fig 2B).

To dissect the relationship between the *abaA*-specific genes and conidiogenesis, we focused on downregulated genes in the *abaA* mutant (WT-1 d/*abaA*-1 d > 3, 933 genes) and categorized them into 10 groups depending on their expression patterns during conidiation (S2 Table and S1 Fig). Genes included in clusters 3 (239 genes) and 9 (125 genes) showed expression patterns similar to *WETA* and *ABAA*, respectively (Fig 2C). We hypothesized that genes under control of WetA or AbaA were members of these groups. Intriguingly, most genes in clusters 3 and 9 (264/364) were upregulated during the initial stage of perithecia development (Fig 2D). These results suggest that many of the genes required for asexual reproduction but not sexual development were highly expressed after sexual induction in *F*. *graminearum*.

**Fig 3:**
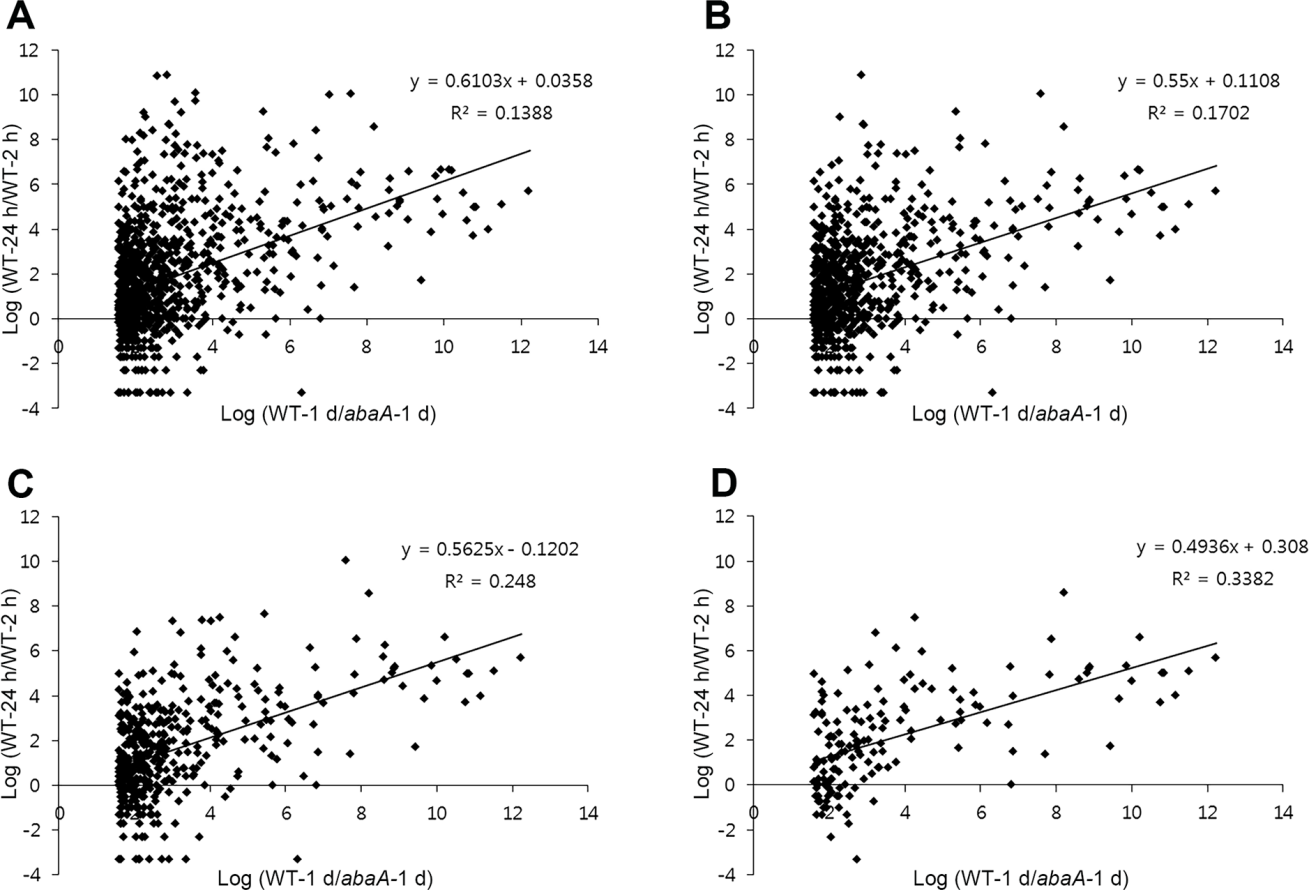
Correlation analyses of transcriptomes depending on expression levels. Genes with more than 0 (A), 1 (B), 10 (C), and 100 (D) RPKM values 12 h after conidia induction were selected for the analysis.

**Fig 4:**
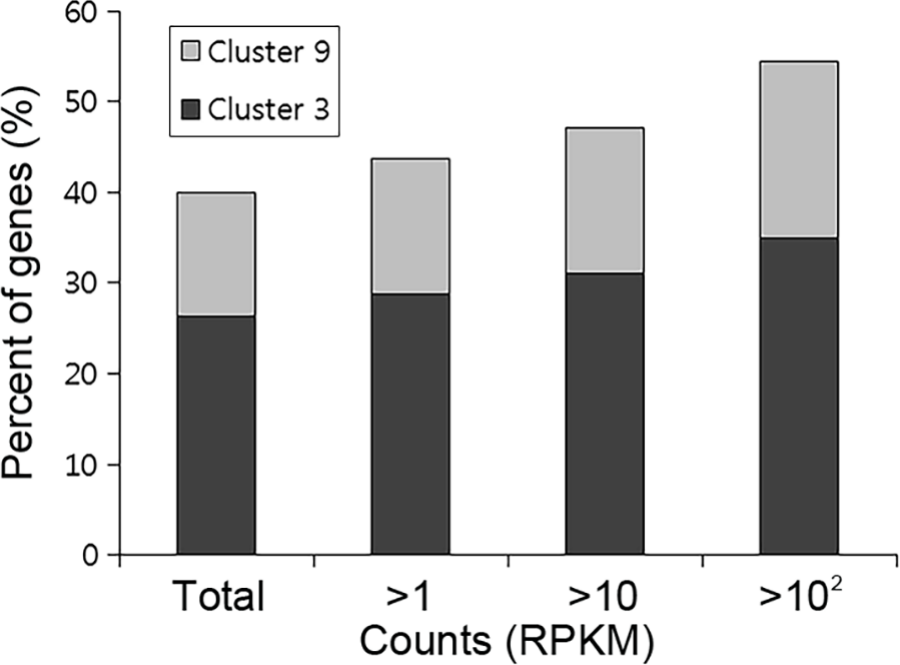
Percentages of genes included in clusters 3 and 9 based on their expression levels. Genes with more than 1, 10, and 100 RPKM values 1 d after sexual induction were selected for the analysis.

Genome-wide functional analysis of transcription factors revealed the distinct positive correlation between the expression level and biological significance in *F*. *graminearum* [15]. We investigated the genetic correlation of fold changes between conidia-deficient and sexually-induced conditions based on the gene expression levels. First, correlations of fold changes between two experimental sets (WT-24 h/WT-2 h and WT-1 d/*abaA*-1 d) are shown as dot graphs (Fig 3). We focused on downregulated genes in the *abaA* mutant (WT-1 d/*abaA*-1 d > 3, 933 genes) for this analysis. As the cut-off RPKM values 12 h after conidia induction (0, 1, 10, and 100 for Fig 3A, 3B, 3C and 3D, respectively) were increased, fold changes of the two experimental sets showed stronger positive correlations. Additionally, highly expressed genes clearly tended to occur more frequently in clusters 3 and 9 (Fig 4). Taken together, we concluded that *ABAA* deletion abolished the expression of genes with a higher biological significance for conidiation.

### Verification of the usefulness of the *abaA* mutant for sexual development studies

**Fig 5:**
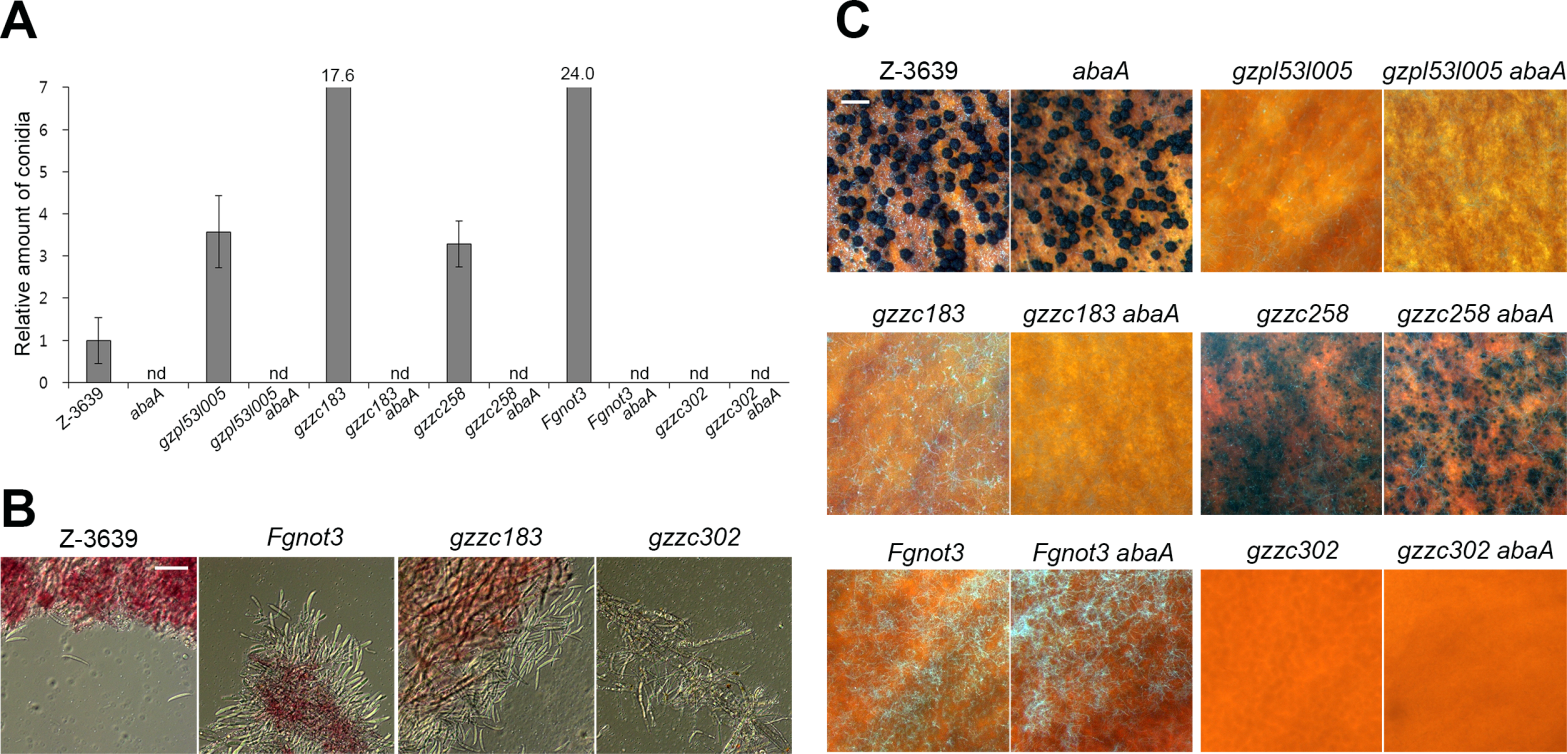
Phenotypic effects of *ABAA* deletion on representative transcription factor mutants. (A) Conidia production. The number of conidia was measured 1 day after sexual induction. The number of conidia in the wild-type strain was arbitrarily set to one. (B) Mycelia and conidia of representative transcription factor mutants. Pictures were taken 1 day after sexual induction. Scale bar = 50 μm. (C) Sexual development of the transcription factor mutant with and without the *ABAA* deletion. Pictures were taken 7 days after sexual induction. Scale bar = 500 μm.

We investigated whether vigorous conidiation also occurred in *F*. *graminearum* perithecia-deficient mutants after sexual induction. We selected five representative transcription factor mutants for this analysis (Table 1). Three mutants (*gzpl53l005*, *gzzc183*, and *gzzc258*) had defects only in perithecia production but not in other phenotypes, whereas two mutants (*Fgnot3* and *gzzc302*) showed pleiotropic developmental defects [15]. Among them, four mutants were normal in conidia production; only the *Fgnot3* mutant produced significantly lower amounts of conidia compared to wild type in liquid medium [15,28]. Four mutants (*gzpl53l005*, *gzzc183*, *gzzc258*, and *Fgnot3*) highly produced conidia compared to wild type, whereas the *gzzc302* mutant did not produce conidia after sexual induction (Fig 5A and 5B). In particular, a huge amount of conidia and conidiophores were observed in the *gzzc183* and *Fgnot3* mutants (Fig 5B). We thought that the variable conidiogenesis phenotypes during sexual development of the null mutants would be major obstacles to obtain quality transcriptomes. Therefore, transcriptome analyses of the perithecia-deficient mutants should be carefully performed to avoid false-positive signals caused by different conidiation phenotypes.

We questioned whether the conidia-deficient mutant *abaA* could be used as the alternative background strain to study sexual development in *F*. *graminearum*. To construct transcription factor mutants carrying the *ABAA* deletion, the Δ*mat2* strain was spermatized with hyphal fragments of Δ*abaA* to generate the HK167 (Δ*mat2* Δ*abaA*) strain (Table 1). Then, the HK167 strain was outcrossed with each transcription factor mutant. Subsequent genotyping confirmed the presence of double deletion mutants carrying deletions of both the transcription factor gene and *ABAA*. As expected, none of the double mutants produced conidia after sexual induction, suggesting that the effect of *ABAA* deletion was epistatic to the effects of the transcription factor genes (Fig 5A). We also examined whether the *ABAA* deletion affected the perithecia development phenotypes. The results confirmed that each single transcription factor mutant and corresponding double deletion mutant carrying the *ABAA* deletion showed indistinguishable perithecia production phenotypes (Fig 5C).

In a previous genome-wide functional analysis on transcription factor genes, we identified 105 mutants that were defective in multiple steps of sexual development in *F*. *graminearum* [15]. Among them, 44 mutants never produced mature perithecia and thus might be useful for studies of the mechanisms underlying the initial stages of perithecia development. Massive comparative transcriptome analyses using transcription factor mutants carrying the *ABAA* deletion will be performed to construct the genetic networks that orchestrate sexual reproduction in *F*. *graminearum*.

## Conclusions

We provided phenotypic and genetic evidence that the current condition used for perithecia induction inevitably highly induced asexual sporulation in *F*. *graminearum*. Our comparative bioinformatics analysis revealed that many genes required for conidiation exhibited altered expression during the initial stages of sexual development. Moreover, deletion of *ABAA* specifically blocked conidia production in both the wild-type strain and sexually-defective mutants during sexual development. We strongly suggest that an alternative background strain (*abaA*) could be used to study the initial stages of perithecia production to obtain high quality transcriptome data.

## Supporting Information

S1 Fig Expression profiles of genes included in the clustered groups. Fuzzy clustering categorized 933 upregulated wild-type-specific genes into 10 groups.
(TIF)

S1 Table Total RNA-seq data used in this study. (XLS)

S2 Table Upregulated genes in the wild-type strain compared to the *abaA* mutant. (XLS)
