# Supplementary figures and images for: Utilization of a Conidia-Deficient Mutant to Study Sexual Development in Fusarium graminearum
Source: PLoS One. 2016 May 13;11(5):e0155671. doi: 10.1371/journal.pone.0155671 (PMC4866773; doi:10.1371/journal.pone.0155671)

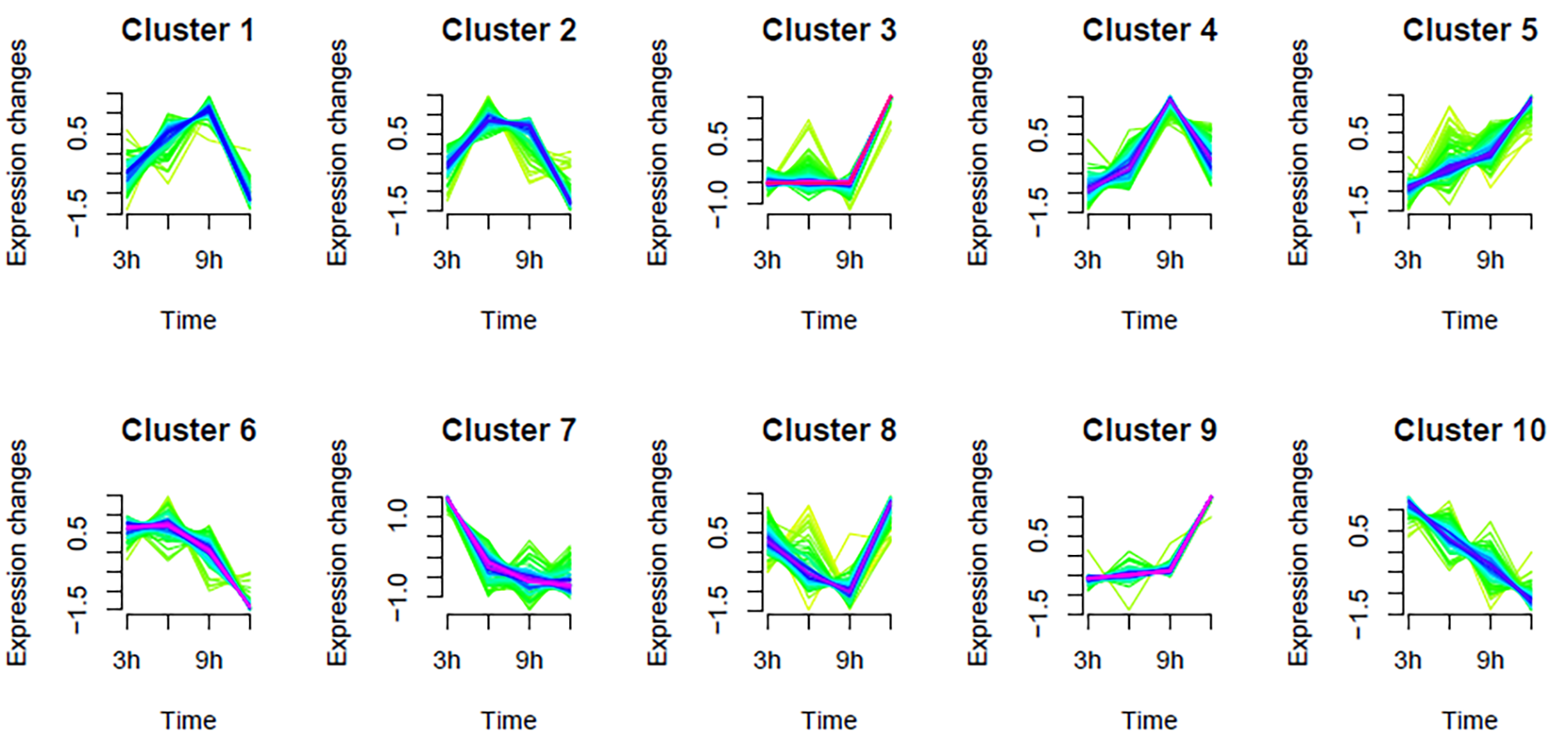

Supplement: S1 Fig — Fuzzy clustering categorized 933 upregulated wild-type-specific genes into 10 groups. (TIF) [file pone.0155671.s001.tif]
